# Evaluation of Knowledge of Immunotherapy Toxicities Among Emergency Physicians in Riyadh, Saudi Arabia

**DOI:** 10.7759/cureus.30325

**Published:** 2022-10-15

**Authors:** Arwa Alahmadi, Haya Altamimi, Mohammed Algarni

**Affiliations:** 1 College of Medicine, King Saud Bin Abdulaziz University for Health Sciences, Riyadh, SAU; 2 Department of Oncology, National Guard Health Affairs, Riyadh, SAU

**Keywords:** immunotherapy toxicity, education, emergency physicians, cancer immunotherapy, knowledge

## Abstract

Introduction

Immunotherapy is considered a new modality in the treatment of cancer with emerging different toxicity profiles. It is essential for healthcare practitioners to be aware of these side effects. Emergency medicine physicians are first-line health providers and should have the required knowledge and understanding of immunotherapy-related adverse effects to be able to identify and manage such patients. The study aimed to assess the level of knowledge of immunotherapy toxicity among emergency medicine physicians in Riyadh.

Methods

This cross-sectional study was conducted at the largest emergency medicine training centers in Riyadh. In total, 106 emergency medicine physicians participated. The questionnaire contained multiple-choice questions that assessed the knowledge and management of immunotherapy-related toxicities.

Results

The majority of the participants were male residents. The response rate varied for the selected training centers. Regarding the level of knowledge regarding the toxic side effects of cancer immunotherapy, the majority were likely to choose “I don’t know.”

Conclusion

This study, in support of the literature, revealed the gap in knowledge of the basic principles of cancer immunotherapy, despite increasing uses and indications of immunotherapy. The findings indicate the need for non-oncologist health practitioners, including emergency physicians, to enhance their knowledge of immunotherapy-related adverse events in order to improve their clinical decision‐making skills.

## Introduction

Cancer treatment methods have changed considerably in recent years, from standard surgical, chemotherapeutic, and radiotherapeutic interventions, to the emergence of targeted therapies, including tumor-specific immunotherapy [1]. Immunotherapy is considered a new modality in the treatment of patients with cancer. However, a new profile of toxicity has emerged [2]. Immunotherapy has complex and variable toxic effects, most are reversible and manageable, and early management will increase the patient's chance of recovery [3]. Immunotherapy enhances the body’s immune defenses against cancer. It uses laboratory-made or natural body products to support, target, or restore the body’s immunocompetence. Some immunotherapeutic agents directly attack the cancer cells and prevent metastasis. Other types boost the immune system to attack cancer cells [4,5]. Immunotherapy includes the use of monoclonal antibodies, cancer vaccination, oncolytic virus therapy, adoptive T-cell therapy, and immune checkpoint inhibitors [6].

Because immunotherapy is evolving as a new modality to treat oncology patients, it is essential for healthcare practitioners to understand its side effects. Immunotherapeutic agents produce a broad profile of toxicity (immune-related adverse effects) requiring specific management [7,8]. Immune checkpoint inhibitor agents (ICIs) are one of the most effective treatments but are linked to toxic side effects. The most affected organs are the skin, lungs, colon, liver, and endocrine organs. Other toxicities are uncommon but could be fatal, for example, neurological and myocardial adverse events [9,10]. The Farooq et al. study reported a global rise in neurotoxic adverse effects (NAE) with ICIs, particularly with the use of dual ICI combinations [11]. NAEs that are associated with the use of ICIs can include peripheral neuropathy, dysgeusia, and paraesthesia. The peripheral nervous system involvement with ICIs can produce mild to moderate peripheral neuropathy or more deleterious adverse effects like Guillain-Barré syndrome, myasthenia gravis, and myositis. However, when comparing NAEs caused by ICIs to NAEs caused by chemotherapy, targeted therapy, vaccines, and combination therapies, the risk is significantly lower with ICIs use. Conversely, patients treated with ICIs therapy had higher rates of insomnia and dizziness [11].

The literature review showed that there is only one published study targeting the level of knowledge related to immunotherapy toxicity among emergency medicine (EM) physicians [12]. Most studies examined the level of knowledge of immunotherapy among multidisciplinary healthcare professionals. The findings were alarming, as there was a lack of knowledge that might prevent the correct treatment of cancer patients [13].

A study conducted in the United States on 150 non-oncology healthcare providers to assess the gaps in immunotherapy-related knowledge, reported a significant gap in the knowledge of cancer immunotherapy, with half of the sample not feeling comfortable treating nor managing cancer immunotherapy patients [14]. A similar cross-sectional study was conducted in the Eastern Province of Saudi Arabia. The study surveyed 360 multidisciplinary healthcare providers and assessed their general knowledge of cancer immunotherapy. The results demonstrated an overall low level of knowledge in the sample, with only 6.4% achieving a high score [15]. Another cross-sectional study interviewed oncologists from the European Union and their findings supported the prior published literature. The study assessed the differences in immunotherapy-related knowledge and practice patterns in oncologists from six European countries; however, toxicity was not one of the items evaluated. The study concluded that only 35% of the oncologists considered themselves knowledgeable about immunotherapy. Most of the sample were more familiar with chemotherapy, radiotherapy, and endocrine therapy and focused more on data related to the clinical practice instead of the mechanism of action and molecular aspects of immunotherapy agents. The sample considered immunotherapy as a research tool, instead of using it in clinical practice [16]. However, a study in the Arabian Gulf countries examined 112 physicians’ understanding of cancer immunotherapies. The sample was knowledgeable about immunotherapeutics, but more than half had little experience in prescribing cancer immunotherapy drugs. The overall results showed a high level of awareness with a mean score of 7.4 [17].

The level of knowledge of immunotherapy-related toxicity in EM physicians is under-researched, both nationally and internationally [18]. EM physicians are the first-line healthcare providers and they should be aware of immunotherapy-related adverse events to enable them to properly identify and manage these patients. Immediate recognition is critical for appropriate management [19-21]. This cross-sectional study aimed to assess and compare the level of scientific knowledge of immunotherapy toxicity and management in EM consultants and residents. The results of this questionnaire-based survey study will provide insight into the level of knowledge of non-oncologist health practitioners. Understanding these aspects will facilitate the promotion of education for non-oncologist health practitioners to improve their clinical decision‐making skills. The ultimate goal and challenge of this study are to ensure that the findings will be disseminated to oncologists to promote educational activities for non-oncologist health practitioners to improve their clinical decision‐making skills of oncology patients and minimize variations in their clinical practice.

## Materials and methods

This study was a cross-sectional study conducted at the EM departments in the seven largest residency training programs in Riyadh, Saudi Arabia. The programs included King Abdulaziz Medical City (KAMC), King Faisal Specialized Hospital (KFSH), King Fahad Medical City (KFMC), King Saud Medical City (KSMC), and Prince Sultan Military Medical City (PSMMC), King Khalid University Hospital (KKUH), and Security Forces Hospital (SFH). The study was conducted between 2019 and 2020.

The data were collected from both male and female non-oncologist residents and consultants from all nationalities in the EM departments in the chosen programs using non-random convenience sampling. A self-developed questionnaire was constructed based on a literature review. An electronic-based questionnaire collecting quantitative data responses with close-ended questions was used. The questionnaire included demographic information, including gender, age, specialty, level of training, and affiliation. Multiple-choice questions were used to assess the level of knowledge related to immunotherapy toxicity and management. The directors of each EM residency program were contacted and they distributed the questionnaires to the EM residents and consultants. The questionnaire was tested for reliability and validity. Internal consistency was assessed by using Cronbach's alpha (α = 0.73). The validity of the questionnaire was tested in a pilot study conducted at KAMC using the same sampling technique. The sample size was 30 physicians and the eligibility criteria for the participants were the same as in the main study. The questionnaire cover letter included a brief explanation of the study and a consent form was completed by each physician.

The validated questionnaire was electronically distributed and collected through the program director of each department. Convenience sampling was used. All analyses were performed using SPSS (IBM Corp., Armonk, NY). The data did not have a normal distribution, and non-parametric tests were used for analysis. The knowledge scores are presented as a median and interquartile range (IQR). The comparison between the knowledge scores and the demographic variables was tested using a Mann-Whitney U test and Kruskal Wallis tests. The distribution was skewed and tested with the Shapiro-Wilks test. A p-value of <0.05 was considered significant. The Institutional Review Board (IRB) of King Abdullah International Medical Research Center approved the study (IRB approval number SP19/079/R). Confidentiality and anonymity were maintained throughout the study, and no name or badge number was collected from participants. Only the research team had access to the data during the study and after completion.

## Results

Demographic characteristics of the sample

From Table 1, the majority of the participants (65.1%) were male and 67% were in the 20-30-year age group. The sample was selected from the largest residency training centers in Riyadh, Saudi Arabia. The response rate varied and KFMC included the highest proportion (30.2%). The rest of the sample was from National Guard Health Affair (NGHA), KSMC, KKUH, PSMMC, KFSH, and Security Forces Hospital. EM residents constituted the majority (67%) of the sample. Regarding work experience, the highest proportion (37.7%) had been practicing for less than two years. 

**Table 1:** Demographic Characteristics of the Sample (n=106) NGHA: National Guard Health Affair; KFSH: King Faisal Specialized Hospital; KFSH RC: King Faisal Specialist Hospital & Research Center; KFMC: King Fahad Medical City; PSMMC: Prince Sultan Military Medical City; KSMC: King Saud Medical City; KKUH: King Khalid University Hospital

Category	Variable	Frequency (n)	Percentage (%)
Gender	Male	69	65.1
	Female	37	34.9
Age in years	20-30	71	67
	31-40	21	19.8
	>40	14	13.2
Work experience	<2	40	37.7
	2-5	36	34
	>5	30	28.3
Job title	Consultant	35	33
	Resident	71	67
Affiliation	NGHA	28	26.4
	KFSH RC	6	5.7
	KFMC	32	30.2
	PSMMC	9	8.5
	KSMC	13	12.3
	KKUH	9	8.5
	Security Forces Hospital	5	4.7
	Others	4	3.8

Knowledge of immunotherapy toxicity in the sample

The level of knowledge regarding immunotherapy toxicity was assessed with an electronic-based questionnaire (n=106). A summary of the findings is presented as frequency and percentage in Table 2. General background knowledge of immunotherapy is presented in Table 2 - Section A, side effects of immunotherapy (Table 2 - Section B), and management of immunotherapy side effects (Table 2 - Section C). We also assessed the willingness of EM physicians to attend educational activities to improve their understanding of this clinical area (Table 2 - Section D).

**Table 2:** Assessment of EM Physicians' Knowledge of Immunotherapy Toxicities EM: emergency medicine

Question	Option	Frequency (%)		Total N(%)
		Consultants	Residents	
	Section A. General Background of Immunotherapy			
1. Do you encounter cancer patients during working hours?	Yes	34 (97.1)	69 (97.2)	103 (97.2)
	No	1 (2.9)	2 (2.8)	3 (2.8)
2. How do you encounter these cancer patients?	Emergency visit or admission	1 (2.9)	2 (2.8)	3 (2.8)
	Inpatients rounds	33 (94.3)	68 (95.8)	101 (95.3)
	Consultation/referrals	-	1 (1.4)	1 (0.9)
	Outpatient clinic	1 (2.9)	-	1 (0.9)
	Other	-	-	-
3. How often are you exposed to cancer patients?	Daily	13 (37.1)	27 (38)	40 (37.7)
	Weekly	8 (22.9)	25 (35.2)	33 (31.1)
	Monthly	7 (20.0)	7 (9.9)	14 (13.2)
	Every couple of months	6 (17.1)	9 (12.7)	15 (14.2)
	During on calls	1 (2.9)	3 (4.2)	4 (3.8)
4. Which one of the following is true about cancer immunotherapy?	It is chemotherapy	4 (11.4)	4 (5.6)	8 (7.5)
	It is a special form of radiation treatment	0 (0)	1 (1.4)	1 (0.9)
	It is a hormonal therapy	0 (0)	3 (4.2)	3 (2.8)
	It is an immune checkpoint inhibitor	26 (74.3)	49 (69.0)	75 (70.8)
	Other	0 (0)	1 (1.4)	1 (0.9)
	I don’t know	5 (14.3)	13 (18.3)	18 (17)
5. Cancer immunotherapy is prescribed by:	Immunologist	4 (11.4)	9 (12.7)	13 (12.3)
	Medical oncologist	17 (48.6)	42 (59.2)	59 (55.7)
	Radiation oncologist	0 (0)	4 (5.6)	4 (3.8)
	I don't know	14 (40)	16 (22.5)	30 (28.3)
6. In clinical practice, how often immunotherapies are used:	Rarely used in clinical practice	2 (5.7)	5 (7)	7 (6.6)
	Daily	3 (8.6)	7 (9.9)	10(9.4)
	Weekly	6 (17.1)	11 (15.5)	17 (16)
	Monthly	13 (37.1)	20 (28.2)	33 (31.1)
	I don't know	11 (31.4)	28 (39.4)	39 (36.8)
7. in clinical practice, in which setting cancer immunotherapy is usually given:	In outpatient setting	6 (17.1)	13 (18.3)	19 (17.9)
	In inpatient setting	12 (34.3)	21 (29.6)	33 (31.1)
	In ICU with close monitoring	0 (0)	2 (2.8)	2 (1.9)
	I don't know	17 (48.6)	35 (49.3)	52 (49.1)
8. In clinical practice: cancer immunotherapy is used more frequently in which of the following:	Lung cancer	7 (20)	15 (21.1)	22 (20.8)
	Brain tumors	1 (2.9)	3 (4.2)	4 (3.8)
	Breast cancer	6 (17.1)	11 (15.5)	17 (16)
	Ovarian cancer	3 (8.6)	4 (5.6)	7 (6.6)
	I don't know	18 (51.4)	38 (53.5)	56 (52.8)
	Section B. Side Effects of Immunotherapy			
1. The side effect of cancer immunotherapy happens:	After multiple doses of treatment	10 (28.6)	7 (9.9)	17 (16)
	Unlikely to happen after the first dose	1 (2.9)	1 (1.4)	2 (1.9)
	It can happen any time during treatment	16 (45.7)	36 (50.7)	52 (49.1)
	I don’t know	8 (22.9)	27 (38)	35 (33)
2. Common minor side effects of cancer immunotherapy are:	Skin Rash	17 (48.6)	32 (45.1)	49 (46.2)
	Asymptomatic Hypercalcemia	0 (0)	2 (2.8)	2 (1.9)
	Myelosuppression	4 (11.4)	7 (9.9)	11 (10.4)
	I don’t know	14 (40)	29 (40.8)	43 (40.6)
3. Which of the following organ is commonly affected by cancer immunotherapy:	Kidney	11 (31.4)	17 (23.9)	28 (26.4)
	Thyroid	8 (22.9)	13 (18.3)	21 (19.8)
	Adrenal	1 (2.9)	1 (1.4)	2 (1.9)
	Liver	2 (5.7)	5 (7)	7 (6.6)
	I don’t know	13 (37.1)	35 (49.3)	48 (45.3)
4. Life-threatening side effects of cancer immunotherapy include:	Pneumonitis	7 (20)	22 (31)	29 (27.4)
	Tumour Lysis syndrome	10 (28.6)	10 (41.1)	20 (18.9)
	Febrile neutropenia	9 (25.7)	13 (18.3)	22 (20.8)
	I don't know	9 (25.7)	26 (36.6)	35 (33)
5. Cancer immunotherapy toxicity to the thyroid gland, include:	Asymptomatic abnormalities	1 (3.7)	3 (6.7)	4 (5.6)
	Thyroid storm	11 (40.7)	9 (20)	20 (27.8)
	Hypothyroidism	3 (11.1)	4 (8.9)	7 (9.7)
	The thyroid gland less likely to be affected	1 (3.7)	0 (0)	1 (1.4)
	I don’t know	11 (40.7)	29 (64.4)	40 (55.6)
	Section C. Management of Immunotherapy Toxicity			
1. Do you feel comfortable to manage the side effects of cancer immunotherapy in the ER setting?	Yes	12 (34.3)	31 (43.7)	43 (40.6)
	No	23 (65.7)	40 (56.3)	63 (59.4)
2. In your practice, do you use common terminology criteria for adverse events (CTCAE):	Yes	7 (20)	7 (9.9)	14 (13.2)
	No	8 (22.9)	23 (32.4)	31 (29.2)
	I don't know this reference	20 (57.1)	41 (57.7)	61 (57.5)
3. As a general approach: the management of cancer immunotherapy side effects	Requires admission	13 (37.1)	27 (38.0)	40 (37.7)
	Steroids are the mainstay of treatment	8 (22.9)	10 (14.1)	18 (17.0)
	Early recognition of toxicity has not been shown to improve outcomes	3 (8.6)	4 (5.6)	7 (6.6)
	Discussion/referral to the medical oncologist should be reserved for patients requiring admission	11 (31.4)	30 (42.3)	41 (38.7)
4. Management of mild rash and pruritus related to cancer immunotherapy	Topical corticosteroid creams	14 (40)	27 (38)	41 (38.7)
	Oral antibiotic	5 (14.3)	1 (1.4)	6 (5.7)
	Admission for observation	2 (5.7)	10 (14.1)	12 (11.3)
	I don’t know	14 (40)	33 (46.5)	47 (44.3)
5. If pneumonitis is suspected in patient receiving cancer immunotherapy:	PFT is the diagnostic test	2 (5.7)	3 (4.2)	5 (4.7)
	A high-resolution CT scan is required	18 (51.4)	29 (40.8)	47 (44.3)
	Bronchoscopy is contraindicated	3 (8.6)	2 (2.8)	5 (4.7)
	I don't know	12 (34.3)	37 (52.1)	49 (46.2)
6. Management of immunotherapy-associated diarrhea includes:	Checking for the underlying cause of diarrhea such as infection	14 (40)	28 (39.4)	42 (39.6)
	Oral or intravenous corticosteroids must be started empirically	3 (8.6)	12 (16.9)	15 (14.2)
	Colitis is less likely to be associated with cancer immunotherapy	0 (0)	1 (1.4)	1 (0.9)
	I don't know	18 (51.4)	30 (42.3)	48 (45.3)
7. If pneumonitis is suspected in a patient receiving immunotherapy	Corticosteroids should be started	11 (31.4)	23 (32.4)	34 (32.1)
	Antibiotics must be given with steroids	8 (22.9)	14 (19.7)	22 (20.8)
	I don’t know	16 (45.7)	34 (47.9)	50 (47.2)
8. The recommended starting dose of prednisone for severe colitis or pneumonitis is:	1-2 mg /kg	14 (40)	13 (18.3)	27 (25.5)
	2-3 mg /kg	3 (8.6)	9 (12.7)	12 (11.3)
	60 mg flat dose	1 (2.9)	4 (5.6)	5 (4.7)
	I don't know	17 (48.6)	45 (63.4)	62 (58.5)
	Education			
1. Did you receive any education about cancer immunotherapy before?	Yes	2 (5.7)	9 (12.7)	11 (10.4)
	No	33 (94.3)	62 (87.3)	95 (89.6)
2. Section D. Do you think awareness about immunotherapy is relevant to your specialty training?	Yes	32 (91.4)	56 (78.9)	88 (83)
	No	3 (8.6)	15 (21.1)	18 (17)
3. Are you willing to attend educational activities related to cancer immunotherapy?	Yes	23 (65.7)	54 (76.1)	77 (72.6)
	No	12 (34.3)	17 (23.9)	29 (27.4)
Total		35 (100%)	71 (100)	106 (100)

Several similarities in the level of knowledge of the general background of cancer immunotherapy occurred in the sample. The majority (74.3%) of the EM consultants knew that immunotherapy was an immune checkpoint inhibitor, compared to 69% of the EM residents. A summary of the sample’s knowledge regarding the general background of cancer immunotherapy is presented in Table 2 - Section A. Regarding the level of knowledge related to the toxic side effects of cancer immunotherapy, the majority of the sample chose “I don’t know” (Table 2 - Section B).

Only 40.6% of the sample considered themselves comfortable to manage the adverse effects of cancer immunotherapy. We assessed their use of the Common Terminology Criteria for Adverse Events (CTCAE), and 87% reported that they were not familiar with this reference nor used it in their clinical practice. Only a small proportion (17%) knew that steroid therapy was the mainstay of treatment for cancer immunotherapy side effects, and only 25% knew the correct starting dose of prednisone. A summary of the sample’s knowledge regarding the management of immunotherapy toxicity is presented in Table 2 - Section C.

In terms of prior formal education regarding cancer immunotherapy and their willingness to attend educational activities, 89.6% of the sample received no previous education and 72.6% were willing to attend educational activities. A small proportion (17%) pointed out that this clinical area was not relevant to their practice in the ED (Table 2 - Section D).

Comparing the difference in the knowledge scores

The median and IQR for the knowledge score was 5, ranging from 3 to 7. The comparison between the knowledge scores and the demographic variables was tested using the Mann-Whitney U test and Kruskal Wallis tests. The distribution was skewed and was tested using the Shapiro-Wilks test with a p-value of <0.05. A summary of differences in the scores is presented in Table 3. None of the variables, age, gender, work experience, and job position, were statistically significant. The knowledge scores were higher in females (55.54), consultants (54.26), the age group of 31-40 years (56.43), and the group with more work experience (56.73).

**Table 3:** Comparing the Differences in Knowledge Scores with Gender, Age, Work Experience, and Job Title

Category	Variable	No	Mean Rank	Median & (IQR)	Statistical Test Used & Test Statistic	P-value
Gender	Male	69	52.41	5 (3,7)	Mann-Whitney U test	0.615
	Female	37	55.54	6 (3.5,7)	Mann-Whitney U = 1201.0	
Age in years	20-30	71	53.16	5 (3,7)	Kruskal-Wallis H test, chi-square = 0.309	0.857
	31-40	21	56.43	5 (3,8)		
	>40	14	50.82	5 (3,7)		
Work experience (in years)	<2	40	55.08	6 (4,7)	Kruskal Wallis H test, chi-square = 1.204	0.548
	2-5	36	49.06	5 (3,6)		
	>5	30	56.73	6 (3,8)		
Job title	Consultant	35	54.26	5 (3,8)	Mann-Whitney U test, Mann Whitney U = 1216.0	0.858
	Resident	71	53.13	5 (4,7)		

Exposure to cancer patients

**Figure 1:**
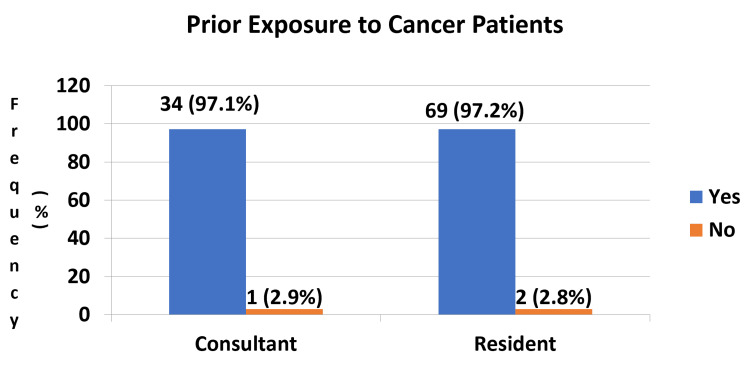
Bar Diagram Demonstrating Prior Exposure to Cancer Patients During Working Hours

**Figure 2:**
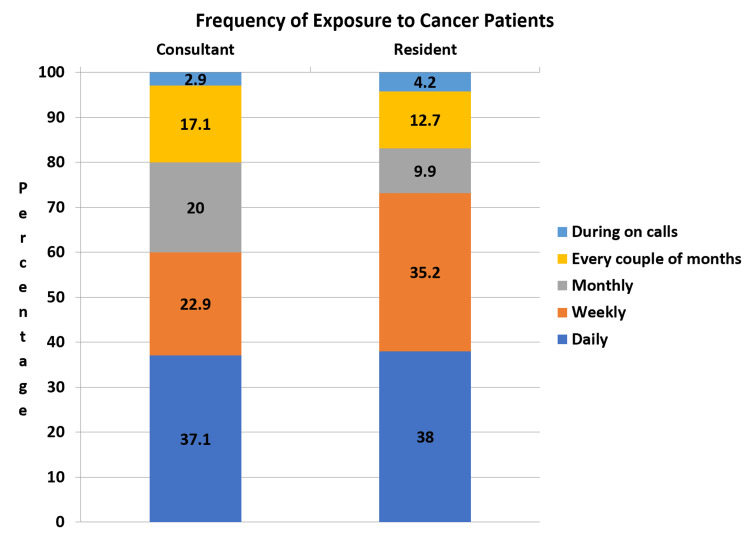
Frequency of Exposure to Cancer Patients

We interviewed emergency physicians in Riyadh for their prior exposure and frequency of exposure to cancer patients during their clinical practice. From Figure 1, the majority of consultants (97.1%) and residents (97.2%) have previously worked with cancer patients. Figure 2 demonstrates that most EM consultants and residents in Riyadh are exposed to cancer patients on a daily basis, 37.1% and 38%, respectively.

## Discussion

The three main domains that were assessed included general background, side effects, and basic management of cancer immunotherapy toxicity. In addition, a minor domain, the willingness to attend educational activities, was also assessed. Our findings indicate an overall low level of knowledge regarding cancer immunotherapy toxicity among emergency physicians in Riyadh. This could be attributable to a lack of education on the matter among junior EM physicians during their residency training and undergraduate programs, as having a longer work experience has resulted in higher levels of knowledge and understanding of immunotherapy toxicity.

The side effects of cancer immunotherapy are potentially serious [3,4,6,8]. EM physicians are the first-line healthcare providers and must have a thorough knowledge of immunotherapy-related adverse effects to enable the identification and management of such patients, as immediate recognition is critical for successful management [21]. This current study revealed a substantial deficit in the level of knowledge of the adverse effects of cancer immunotherapy. as more than half of the participants had a low level of knowledge of cancer immunotherapy side effects. The majority of the participants had inadequate knowledge of the basic principles for the management of immunotherapy toxicities. Only 17% knew that steroid therapy was the mainstay of treatment, and only 25% knew the correct starting dose of prednisone. Although the interviewed sample felt comfortable managing the adverse effects of cancer immunotherapy in ER settings, the scores, as a whole, revealed a significant gap in knowledge.

The level of knowledge regarding the use of the Common Terminology Criteria for Adverse Events (CTCAE) was alarmingly low. CTCAE is an established criterion used for the standard classification of the severity of organ toxicity caused by drugs used in cancer therapy. Eighty-seven percent (87%) of the sample reported they had no prior knowledge of CTACE, nor used it in their clinical practice. An educational activity should help increase awareness of this important tool.

The length of work experience was associated with higher knowledge scores, as evidenced by the lower level of knowledge of cancer immunotherapy of EM residents as compared to EM consultants. The EM consultants had longer work experience, hence they were exposed to more cancer patients and novel immunotherapies in their clinical practice. This finding was similar among the different training program centers in Riyadh though it's not statistically significant. The female gender was associated with higher knowledge scores. Despite the fact that female participants constituted only a third of participants, they had higher levels of knowledge compared to their male counterparts. However, this finding was not consistent with the findings in the literature [15].

The result of the current study supports the known literature, as a study conducted in 2018 on non-oncology healthcare providers in the United States revealed a significant gap in the knowledge of cancer immunotherapy in non-oncology healthcare providers [14]. A study with similar results was also published in early 2020 in the Eastern province of Saudi Arabia [15]. The study surveyed multidisciplinary healthcare providers who also showed a low level of knowledge. Another study with oncologists from the European Union (2014) reported similar findings [16]. These findings are alarming, and the insufficiency of understanding of cancer immunotherapy may affect the proper treatment of cancer patients. One study reported contradicting results; this study, conducted in 2018 on oncologists in the Arabian Gulf countries, reported a high level of understanding; however, there was little experience in prescribing cancer immunotherapy drugs [17]. This study was conducted among oncologists, who are more likely expected to know more about immunotherapy.

To our knowledge, this study is the first to address this issue among EM physicians in Riyadh, Saudi Arabia. It highlights the importance of conducting similar studies in other hospitals in the Kingdom to evaluate the knowledge and practices of EM physicians. However, there were some limitations to our study. The data were collected at a single point in time, and there was some difficulty in the collection process. A major limitation was the coronavirus disease 2019 (COVID-19) pandemic that overwhelmed EM physicians, resulting in a low response rate. The electronic questionnaire used the multiple-choice question (MCQ) format, which may have caused some bias. As a result of collecting the data from only seven major hospitals in one geographical area, the results may not be generalized to the general population of Saudi Arabia. Despite these limitations, we have shown that there is a knowledge gap in immunotherapy-related toxicities among ER physicians. Educational activities are essential to increase awareness, given the willingness of the study participants to be involved in educational activities.

## Conclusions

The current study revealed an alarming lack of knowledge of the basic principles of cancer immunotherapy. Immuno-oncology is constantly advancing, and as a result, the deficit in knowledge will increase over time. Our findings indicate the need for non-oncologist health practitioners, including EM physicians, to enhance their knowledge of the basic principles of cancer immunotherapy. Understanding this will support promoting education to improve their clinical decision‐making skills. The findings of this study should be reported to oncologists to facilitate educational interventions for non-oncologist health practitioners to minimize the variations in their clinical practice.

## References

[1] Olsen TA, Zhuang TZ, Caulfield S (2022). Advances in knowledge and management of immune-related adverse events in cancer immunotherapy. Front Endocrinol (Lausanne).

[2] Gumusay O, Callan J, Rugo HS (2022). Immunotherapy toxicity: identification and management. Breast Cancer Res Treat.

[3] Gangadhar TC, Vonderheide RH (2014). Mitigating the toxic effects of anticancer immunotherapy. Nat Rev Clin Oncol.

[4] Zhang H, Chen J (2018). Current status and future directions of cancer immunotherapy. J Cancer.

[5] Sosa A, Lopez Cadena E, Simon Olive C, Karachaliou N, Rosell R (2018). Clinical assessment of immune-related adverse events. Ther Adv Med Oncol.

[6] Meiliana A, Dewi N, Wijaya A (2016). Cancer immunotherapy: a review. Indones Biomed J.

[7] Conroy M, Naidoo J (2022). Immune-related adverse events and the balancing act of immunotherapy. Nat Commun.

[8] Cousin S, Seneschal J, Italiano A (2018). Toxicity profiles of immunotherapy. Pharmacol Ther.

[9] Wang DY, Salem JE, Cohen JV (2018). Fatal toxic effects associated with immune checkpoint inhibitors: a systematic review and meta-analysis. JAMA Oncol.

[10] Haanen JB, Carbonnel F, Robert C, Kerr KM, Peters S, Larkin J, Jordan K (2017). Management of toxicities from immunotherapy: ESMO Clinical Practice Guidelines for diagnosis, treatment and follow-up. Ann Oncol.

[11] Farooq MZ, Aqeel SB, Lingamaneni P (2022). Association of immune checkpoint inhibitors with neurologic adverse events: a systematic review and meta-analysis. JAMA Netw Open.

[12] Butters T, Szabados B, Grant M, Liu W-K, Lam J, Powles T (2019). Do emergency department physicians understand immunotherapy?: survey in the largest London university hospital trust. Eur Urol Suppl.

[13] Simmons D, Lang E (2017). The most recent oncologic emergency: what emergency physicians need to know about the potential complications of immune checkpoint inhibitors. Cureus.

[14] (2018). Identifying gaps in immunotherapy education beyond the oncology team. https://www.accc-cancer.org/docs/immuno-oncology/abstracts-and-presentations/mascc-2018-identifying-gaps.pdf?sfvrsn=375e214b_0.

[15] Hamad RS (2020). Evaluation of awareness and understanding of cancer immunotherapy among healthcare professionals in eastern Saudi Arabia. J Oncol Pharm Pract.

[16] Borrello IM, Schaffer MM, Roehrl E, Marshall JF (2014). Identification of differences in immunotherapy knowledge and practice patterns among oncologists from six European countries. Mol Clin Oncol.

[17] Al-Shamsi HO, Tashkandi E, Bukhari N (2018). Awareness, understanding, attitude and barriers toward prescribing modern cancer immunotherapies in the Arabian Gulf countries. Gulf J Oncolog.

[18] Butters T, Liu WK, Grant M (2021). What do emergency physicians know about immune checkpoint inhibitor-related toxicities: a brief report. Int J Cancer Manag.

[19] Hsiehchen D, Watters MK, Lu R, Xie Y, Gerber DE (2019). Variation in the assessment of immune-related adverse event occurrence, grade, and timing in patients receiving immune checkpoint inhibitors. JAMA Netw Open.

[20] Herrmann T, Pearce F, Warren C, Kadkhoda H, Wiggins L, Rindo A (2015). Identification of continued challenges and opportunities in oncologists' comprehension of immuno-oncology. J Immunother Cancer.

[21] Sears CR, Peikert T, Possick JD (2019). Knowledge gaps and research priorities in immune checkpoint inhibitor-related pneumonitis. An Official American Thoracic Society research statement. Am J Respir Crit Care Med.

